# Cognitive control adjustments are dependent on the level of conflict

**DOI:** 10.1038/s41598-024-53090-4

**Published:** 2024-02-07

**Authors:** Miklos Bognar, Zsuzsa Szekely, Marton A. Varga, Kinga Nagy, Giacomo Spinelli, Andree Hartanto, Nadyanna M. Majeed, Nicole Rui Ying Chen, Mate Gyurkovics, Balazs Aczel

**Affiliations:** 1 Doctoral School of Psychology, ELTE Eotvos Lorand University, Budapest, Hungary; 2 Institute of Psychology, ELTE Eotvos Lorand University, Budapest, Hungary; 3 Università degli Studi di Milano-Bicocca, Milano, Italy; 4 Singapore Management University, Singapore, Singapore; 5 National University of Singapore, Singapore, Singapore; 6 Institute of Neuroscience and Psychology, University of Glasgow, Glasgow, UK

**Keywords:** Psychology, Human behaviour

## Abstract

The congruency sequence effect (CSE) is one of the most investigated effects in the cognitive control literature. The conflict monitoring theory suggests that the CSE is the result of adjustments in cognitive control based on perceived conflict. A recent paper by Zhang and colleagues, has investigated whether the manipulation of conflict level by changing distractor incompatibility in a flanker task affects the amount of adjustments in cognitive control. Their study produced mixed findings, somewhat supporting the original conflict monitoring theory, but also suggesting other explanations, such as the repetition expectancy account. We replicated the experimental design in a multisite online study (N = 347), with Hungarian, Italian, and Singaporean participants. Our results supported the prediction that changes in the level of conflict trigger conflict adaptation, revealing that increasing conflict levels induced stronger adaptive control. Bayesian hypothesis testing indicated a monotonic reduction in congruency effects as a function of previous conflict strength. This finding is in line with the extension of the traditional conflict monitoring theory, as well as other theories like affective signaling and expected value of control, implying that the relationship between conflict and interference effects is gradual, rather than a binary function.

## Introduction

Cognitive control is a set of mechanisms that help us behave in a goal-oriented fashion in complex or conflicting circumstances. It is responsible for differentiating between controlled and automatic states and maintaining goal-oriented behaviour in the face of otherwise more habitual automatic behaviours^1^. To study cognitive control, researchers often use sequential conflict tasks, where participants need to respond to congruent and incongruent visual stimuli. In a flanker task^2^, an odd number of symbols are presented, and participants are asked to respond to the center symbol while ignoring the flanking symbols. A flanker stimulus thereby can be congruent, when the target stimulus and the flankers are identical (e.g., HHHHH), or incongruent, when the target and the flankers are different (e.g., NNHNN). A well-documented phenomenon in sequential conflict tasks is that responses are slower and less accurate on incongruent trials, compared to congruent trials. This congruency effect however is also dependent on the congruency of previous trials: the congruency effect is smaller following an incongruent trial, compared to a congruent trial^3^. This pattern can be observed in several conflict paradigms and is commonly named the congruency sequence effect^4^ (CSE). The most commonly accepted theory on the CSE is the conflict monitoring theory which suggests that the anterior cingulate cortex monitors the amount of perceived conflict and then the dorsolateral prefrontal cortex adjusts the strength of cognitive control adaptively^5^.

**Figure 1:**
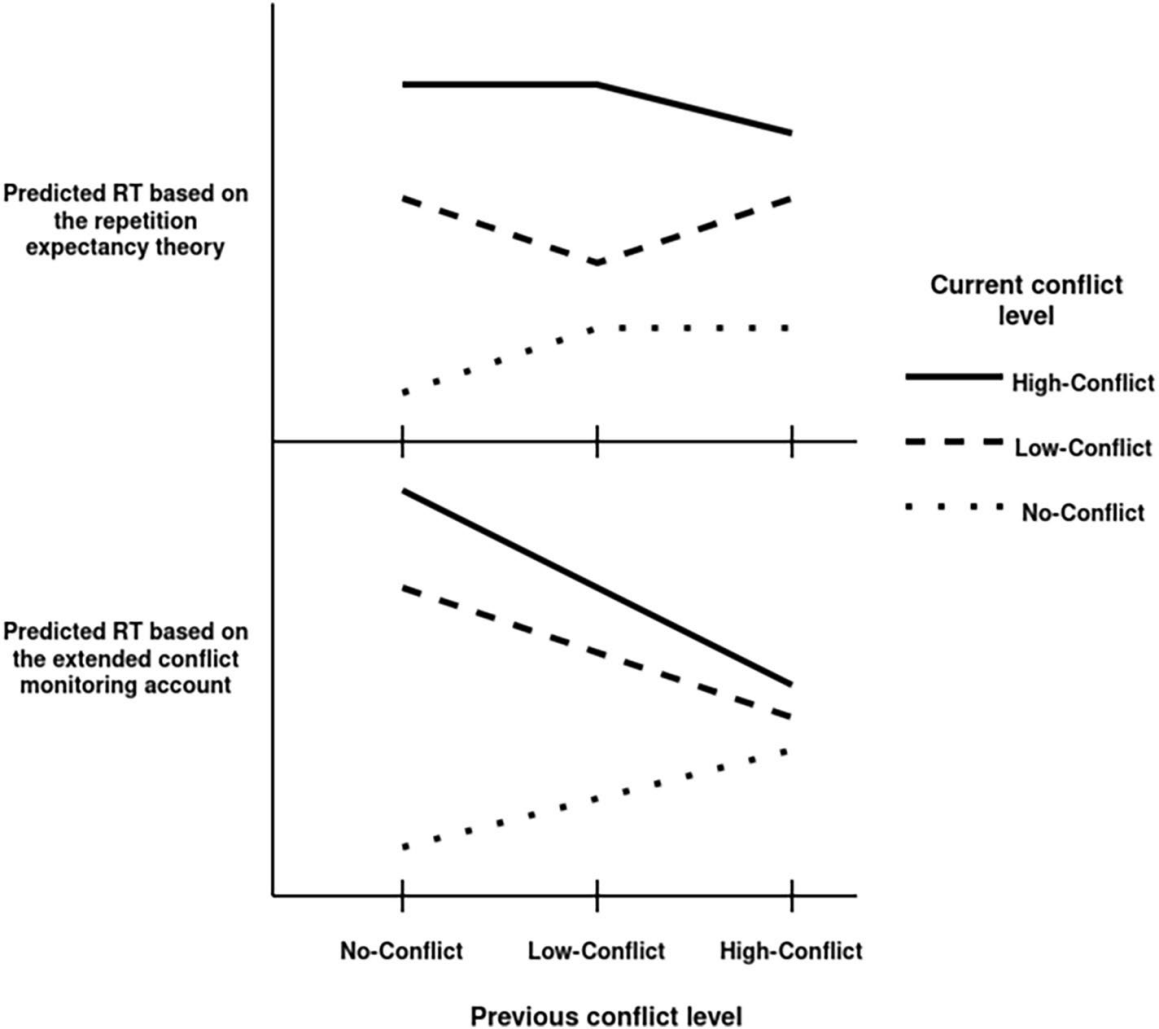
The predicted patterns in RT based on the repetition expectancy and the extended conflict monitoring theories. In the repetition priming model suggested by Zhang and colleagues^12^, conflict type differences are dependent on the expected repetition of the previous conflict level, regardless of the relative conflict level of the previous trial: RTs decrease when the current and previous conflict levels are the same, while increase when current and previous conflict levels are different. In contrast, the extended conflict monitoring account predicts RTs to decrease in current low-, and high-conflict (incongruent) trials with the level of previous conflict, while RT to increase on current no-conflict (congruent) trials with the previous level of conflict.

Based on the conflict monitoring framework and electrophysiological findings^6,7^, an obvious extension of the theory is that the strength of control adaptation depends on the amount of conflict. In this model—consistent with the common interpretation of CSE—reaction time (RT) on incongruent (high-conflict) trials will decrease with the level of previous conflict, due to the reduced interference caused by increased control, whereas RT increases on congruent (no-conflict) trials with previous level of conflict, as the facilitative effects of congruent distractors are decreased. In a framework where the level of induced conflict is not dichotomous—as in traditional congruent-incongruent designs—the extended conflict monitoring theory would predict (1) an increase in RTs with higher levels of conflict and (2) sequential modulation of such conflict effects based on the previous trial’s level of conflict. This prediction might also assume conflict effects in the current trial to be modulated by previous conflict levels in a continuous fashion (see the lower panel of Fig. 1). Forster and colleagues^8^ hypothesized that the congruency effect would decrease according to the level of previous stimulus incompatibility (i.e., the proportion of incongruent flanker letters in a flanker task trial), reflecting a linear relationship between conflict strength and adaptation of control. They found decreased performance on higher distractor levels suggesting that conflict strength can be manipulated by parametrically changing the number of distractors in a trial. Furthermore, they found increased adaptation (smaller congruency effect) on trials preceded by higher levels of stimulus incompatibility, confirming the proposed extended conflict monitoring account. Their design, however, consisted of only two stimulus types and the ratio of congruent and incongruent trials were not equal, therefore their finding could be the product of effects that are considered to be confounds when investigating cognitive control processes with stimulus response incompatibility tasks. Feature integration^9^ refers to the idea that CSE is a product of a binding process that decreases RT in trials where either both the target and the distractor features of previous trials are repeated or neither of them are, situations that typically occur more often for congruent-congruent and incongruent-incongruent sequences, leading to a CSE-like pattern. Contingency learning^10^, another possible confounding effect, occurs when certain targets (most typically, the congruent ones) are more probable than others (most typically, incongruent ones) for a given distractor. Because the facilitating effect of probable vs. improbable target-distractor combinations is larger following probable vs. improbable target-distractor combinations, this sequential contingency effect will also mimic the pattern of CSE. Zhang and colleagues^11^ in their recent study created a design where they separated incongruent trials into low-conflict (LC) and high-conflict trials (HC) and called congruent trials no-conflict (NC) trials, thus creating three levels of conflict instead of a traditional congruent-incongruent dichotomy. This allowed an analysis of sequential congruency (conflict) effects in a 3 × 3 design combining the three levels of conflict in previous and current trials. This design also controlled for the above mentioned confounds and still produced a CSE-like pattern, a pattern that generally supports the extended theory of conflict monitoring.

Zhang and colleagues’ design bears central importance for theoretical reasons. In our everyday life, distracting or conflicting stimuli rarely occur in an all-or-none fashion, but more on a continuous scale, where there are less conflicting and more conflicting distractors in the environment. In the conflict monitoring framework this would mean that the more conflict is detected by the ACC, the more of a cognitive control adjustment will be made to deal with subsequent conflicts. This design allows us to investigate whether conflict strength plays any role in sequential congruency effects, as a binary, or a parametrically manipulable, continuous function.

The authors of the original paper also found ambiguous results that are not in line with the prediction of conflict monitoring: The difference between current low-conflict (LC) and high-conflict (HC) trials was relatively small when they were preceded by no-conflict (NC) or high-conflict trials, while this difference was large when preceded by a low-conflict trial. This pattern was also found in a recent paper^12^ where the same experimental paradigm was replicated, along with the measurement of event-related potentials. They proposed that their finding might provide evidence against the conflict monitoring account, and in favour of the repetition expectation, or repetition priming hypothesis^3,13^, which was the original explanation of the then novel finding of CSE. The hypothesis posits that the CSE is the product of the expectation of repetition in congruency: If the congruency type of the previous trial is repeated in the current trial, RT will decrease, but when it is not repeated, RT increases. Assuming trials differing in conflict levels can be recognized as belonging to no-, low-, and high-conflict types, this model would predict RT to decrease on trials with the same conflict level as the preceding trial, and increased RT on trials that were preceded by a trial with a different conflict level. The model however does not specify that an effect will emerge with partial repetitions of stimulus features, therefore the level of the preceding conflict should not matter when it is different from the current trial’s level (leading, e.g., to equal RTs on current NC trials when preceded by either a LC or a HC trial).

Zhang and colleagues^12^ therefore suggested that it is not the level of conflict strength, but the expected level of conflict that drives adaptation. The repetition priming hypothesis posits that sequential congruency effects are the byproducts of perceptual priming which is a widely investigated memory effect that helps us process repeated stimuli faster^14^. According to this, sequential congruency effects are created by memory processes rather than cognitive control adjustments, however, this dichotomy here might be arbitrary as both can be integrated in an associative learning perspective, which posits that coactivated perceptual, motor, and goal representations in a task form an associative network, coding the overall context, facilitating cognitive control through contextually appropriate associations between perceptual representations and specific goals^15^. The model suggested by Zhang and colleagues^12^, and the above-described extended conflict monitoring framework predict congruency effects to produce different result patterns in the present design (see Fig. 1.).

Some of the contrasts involved in these patterns are somewhat similar. For example, both models predict an RT increase on NC current trials after higher than no-conflict levels in previous trials. The repetition expectancy model explains it by the alternation of conflict types, while conflict monitoring explains it by the decreased facilitation of congruent distractors due to the higher focus on the target. It is important to note, however, that the most prominent difference between the two predictions is in the previous-trial conflict effects on LC current trials (which are monotonic for the extended conflict monitoring model vs. non-monotonic for the repetition expectancy model), thus the testing of these interactions is key to adjudicate between the two models when investigating the effects of parametric manipulation of the level of conflict.

In this study, we attempted to replicate the design of Zhang and colleagues^11^ on a large multisite sample to see whether parametrically changing the level of conflict affects adaptation of control. Our main, preregistered hypothesis was that changing the level of conflict modulates sequential congruency effects. Furthermore, we hypothesized that the level of conflict predicts the strength of control adjustments, and congruency effects (i.e., conflict type differences) will decrease with the increase of conflict level of previous trials. This hypothesis is more stringent than the conclusion of the original paper, which suggested that changes in the level of conflict trigger adaptive control but did not specify the direction of such adaptation. Thus, this replication study also allowed us to investigate which hypothetical model (repetition expectancy or conflict monitoring) predicts the patterns in congruency effects better. To do so, we reported our results in a way that the two models can be compared.

## Methods

### Transparency and open practices

The methods of this study were constructed with the attempt to replicate the design of Zhang and colleagues^11^ as closely as possible. In our endeavour to gather information, we made repeated efforts to establish communication with the original authors; however, our attempts were unsuccessful. Any potential deviations from the initial design in this segment stem from the circumstance that our sole reference for constructing the experiments was the “Methods” section within the original paper. We preregistered this study in 2022, with details about the planned experimental design and analysis plan. The preregistration can be found on Open Science Framework (OSF): https://osf.io/cfjgz. All anonymized data, experiment code, and analysis code can be found on the OSF repository of the study: https://osf.io/e5ryf/

The only major deviation from the methods of the original study was that while the original study was conducted in a laboratory, in this study only Singaporean participants completed the task in laboratory settings. Hungarian and Italian participants completed the experiment online, on their own computer, at home, hence all details that were controlled in the original experiment (e.g. screen resolution, refresh rate, participants’ head eyes distance from the screen) were not controlled for in this study. This deviation from the original study however should not matter much, as several studies have shown that congruency effects and sequential congruency effects are easily reproducible in online settings^16,17^, especially with the flanker task. All minor deviations in the methods are indicated at the corresponding part of the procedure.

### Participants

A total of 347 (73.49% female) participants took part in the experiment. 244 Hungarian undergraduate students from Eötvös Loránd University completed the experiment within the framework of a university course and received course credits for participation. In addition, 47 undergraduate students from University of Milano-Bicocca and 56 undergraduate students from Singapore Management University completed the experiment. The experiment instructions and trial feedback texts were originally written in English, then they were translated into Italian and Hungarian. All sites used language versions according to the official language of the country the sites were located in. For further information about the sampling plan, see the “Sampling plan” section below.

### Ethical approval

Eotvos Lorand University’s institutional ethical review board approves these studies (2022/678). This study was conducted in accordance with the Declaration of Helsinki. Informed consent was obtained from all participants taking part in this study.

### Experimental instruments/materials

The experiment was conducted online on a private server and was implemented in JATOS 3.8^18^, an online experiment management tool. The experiment was coded in jsPsych^19^, a programmable JavaScript framework to design online experiments. This was a minor deviation from the original experiment, which was conducted in E-prime. The modified flanker task consisted of stimuli formed by concatenating horizontally five letters selected among four potential letters: F, H, N, and P. The stimuli were split into two subsets, H–N and F–P (e.g., HHNHH, FFPFF), where the two letters could appear as target, and distractor letters as well. This subsetting of stimuli was used to prevent possible confounds such as contingency learning or feature integration from impacting the sequential congruency effects^20^. The two stimulus subsets alternated from trial to trial. The target letter was flanked by a varying proportion of distractors creating three levels of conflict (e.g., FFFFF—no-conflict, FPPPF—low-conflict, FFPFF—high-conflict). In low-conflict and high-conflict conditions, two and four distractors were different from the target, respectively. In low-conflict, the innermost flanker letters were the same as the target stimulus. All stimuli were presented in the center of the screen in a 40px Arial white font against a black background.

### Task and procedure

**Figure 2:**
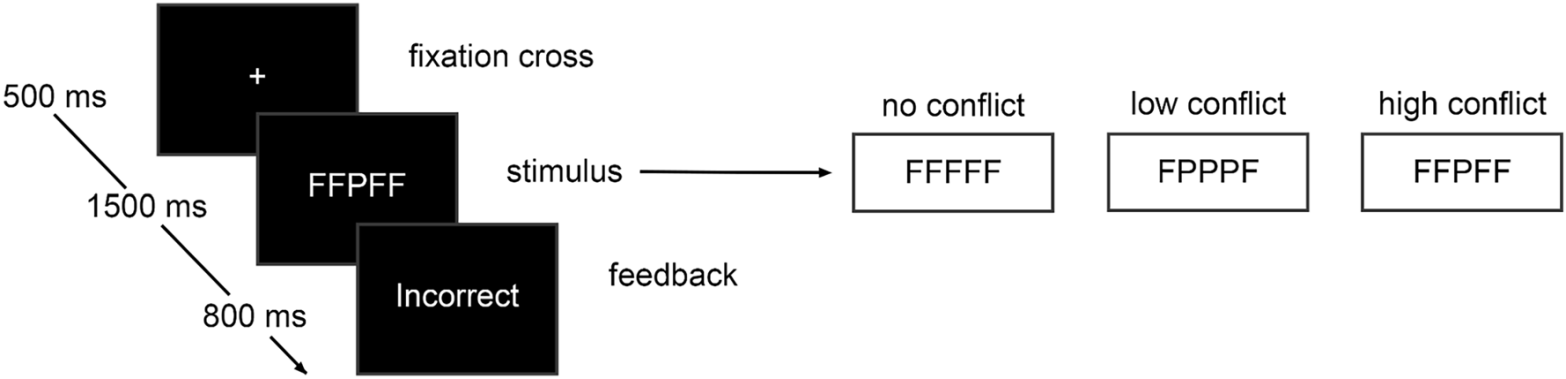
Timings and stimulus types of a trial.

The study involved a 3 (previous trial congruency) × 3 (current trial congruency) within-subject design. Participants completed the same conflict task which consisted of three types of trials regarding the level of conflict between the target and the flanker letters: no-conflict (NC), low-conflict (LC) and high-conflict (HC). The conflict task was made up of a practice block and 6 test blocks. The practice block contained 48 trials (24 NC, 12 LC, 12 HC). The practice block ensured that each participant learned the instructions and maintained accuracy over 85%. If a participant failed to pass this accuracy threshold, the practice block repeated until this threshold was surpassed. The practice block was left out of the analysis. Each test block contained 97 trials, of which 48 NC, 24 LC, and 24 HC were included in the analysis, plus a randomly sampled first trial in the block that was not included in the analysis. This way, congruent (NC) and incongruent (LC and HC) trials were presented in equal proportions. Each trial block had nine types of current–previous trial pairs: NC–NC (24), NC–LC (12), NC–HC (12), LC–NC (12), LC–LC (6), LC–HC (6), HC–NC (12), HC–LC (6) and HC–HC (6). Each stimulus was picked out pseudorandomly according to this distribution. The conflict task procedure (see Fig. 2) started with a grey fixation cross displayed for 500 ms followed by the stimulus. In response to the stimulus, the participants had to press the button corresponding to the target letter while ignoring flanking letters. The corresponding buttons on the computer keyboard were 1 for ‘F’, 2 for ‘H’, 8 for ‘N’, and 9 for ‘P’. The keyboard layout was modified compared to the original experiment (in the original, keys ‘1’,’2’,’9’ and ‘0’ were used), to make sure the same buttons would be pressed on all keyboard layouts between sites. Participants had to use the left middle, left index, right index, and right middle finger, respectively, to respond to those target letters. The stimulus was presented for a maximum time of 1500 ms, or until participant response. A feedback screen following the stimulus was presented for 800 ms and was either blank when the response was correct or read “Incorrect/No response detected” when the response was incorrect or no response had been registered. After feedback, a fixation cross appeared again starting the cycle over that would repeat until the task procedure was completed.

### Statistical approach

In our analyses, we followed the same data exclusion criteria as outlined in the original paper. In addition to the repeated measures ANOVA tests reported in the original paper, in order to quantify raw effect sizes for the Bayes factor estimation, we conducted mixed-effects linear regression analyses. As preregistered, random effects were planned to be participant as random intercept and current conflict level as random slope, however initial analyses showed that the amount of data was not sufficient, therefore models with more complex random effect structures could not converge. To solve this issue, we used only random intercepts in the model. For these additional regression analyses, we calculated Bayes factors (*BF*_10_) that we used to estimate the relative evidence for H1 over H0. We relied on Bayes factors in interpreting the results as they can help us distinguish between evidence for the alternative (or, the null) hypothesis and inconclusive evidence. We used conventional thresholds of 3 and ^1/3^ of *BF*_10_ to discern satisfactory evidence for H1 and H0, respectively. We reported *p*-values alongside the *BF*_10_-s on both models. To calculate Bayes factors, we used the R-script of Dienes and Mclatchie^21^. We constructed models for the predictions of all H1 hypotheses using half-normal or normal distributions centered around zero. This choice was made to represent the possible direction of alternative hypotheses, and the assumption that smaller effects are more likely than larger ones.

We report Bayes factors as *B*_H(*M, SD*)_ or *B*_N(*M, SD*)_ where *B* is the Bayes factor in favour of the alternative hypothesis (B_10_) for a half-normal, or normal prior distribution H or N, with *M* mean and *SD* standard deviation. Prior distributions are described in the “Results” section for every model tested. As a general rule, we used the raw mean congruency effect found in the original paper as the SD of our prior distribution in congruency effect tests, the half of the congruency effect in sequential congruency effects^17^. We also reported the Bayesian Robustness Regions (*RR*) notated as RR[*SD* smallest, *SD* largest] that covers all priors that result in the same conclusion (B > 3, or B < ^1/3^) as the original prior SD^22^. For instance, if the Bayes Factor is higher than 3 with our original prior SD, we also report the highest and the lowest SD that results in a B of 3 or higher. To keep the “Result” section concise, we did not conduct all statistical tests in the original paper, only the tests that were relevant to the theoretical question.

Error rate results in the two studies using this same paradigm were inconsistent^11,12^, but previous examination of classic flanker accuracy also proved to be inconsistent and unreliable with respect to latency effects^20,23^ perhaps due to the task’s overall low difficulty causing a floor effect in error rate. In our analysis we investigated only RTs, as the original paper’s conclusions were solely based on this measure.

To compare the predicted patterns by the two theories we specified interactions between contrasts where the two predictions diverged the most and produced contradicting hypotheses. We used Bayesian analysis with two-tail normal prior distributions allowing for conclusive evidence for both directions in interactions. For more information on the specified interactions, see the “Results” section.

### Data analysis software

Data analysis was fully conducted using the R programming language^24^, version 4.3.1. Several additional R packages were used during the analysis:

tidyverse 2.0^25^; papaja 0.1.1^26^; lme4 1.1^27^; ggdist 3.3^28^; lmerTest 3.1^29^; emmeans 1.8.8^30^; ggeffects 1.3.1^31^; rstatix 0.7.2^32^; BayesFactor 0.9.12^33^; jsonlite 1.8.7^34^; ez 4.4^35^.

To keep analysis code always reproducible, we used the package “groundhog 3.1.1”^36^ with the above listed R packages and the date September 13, 2023.

### Sampling plan and data collection

We followed a Bayesian sequential stopping rule in our sampling plan that allows for additional data collections whenever the Bayesian hypothesis testing result is inconclusive^37^. We chose the most complex interaction effect (previous conflict level × current conflict level on RT) as the reference hypothesis test to decide whether additional data collections were necessary. In the first wave of data collection, we aimed to collect as much data as possible on all sites in the remaining time of the spring semester (approximately 2–3 months, depending on the university’s schedule), but at least 2.5 times the sample size of the original study (N = 31), which is 78 participants. We contacted possible collaborators for a multisite data collection to reach a numerous and diverse sample and agreed on parallel data collections with the University of Milano-Bicocca and the Singapore Management University. We conducted the data collection on all three sites in the spring semester of 2023. As stated above, 347 participants in total completed the test in the first and final round, where the crucial hypothesis test was conclusive, hence no additional data collection was required. The participants from Eötvös Loránd University and University of Milano-Bicocca completed the experiment online individually. Participants from Singapore Management University completed the experiment on-site in a laboratory setting.

## Results

### Data preprocessing

We only analysed test trials, and excluded all trials that were in the practice block. For the RT analysis, we excluded all trials that were the first of their blocks (1.03%), trials that were incorrect or preceded by an incorrect trial (11.71%), and trials with RTs more than 3 standard deviations (SD) from the condition mean (1.07%). In total, 13.56% of all trials were excluded based on these criteria.

### Current trial congruency effects

**Figure 3:**
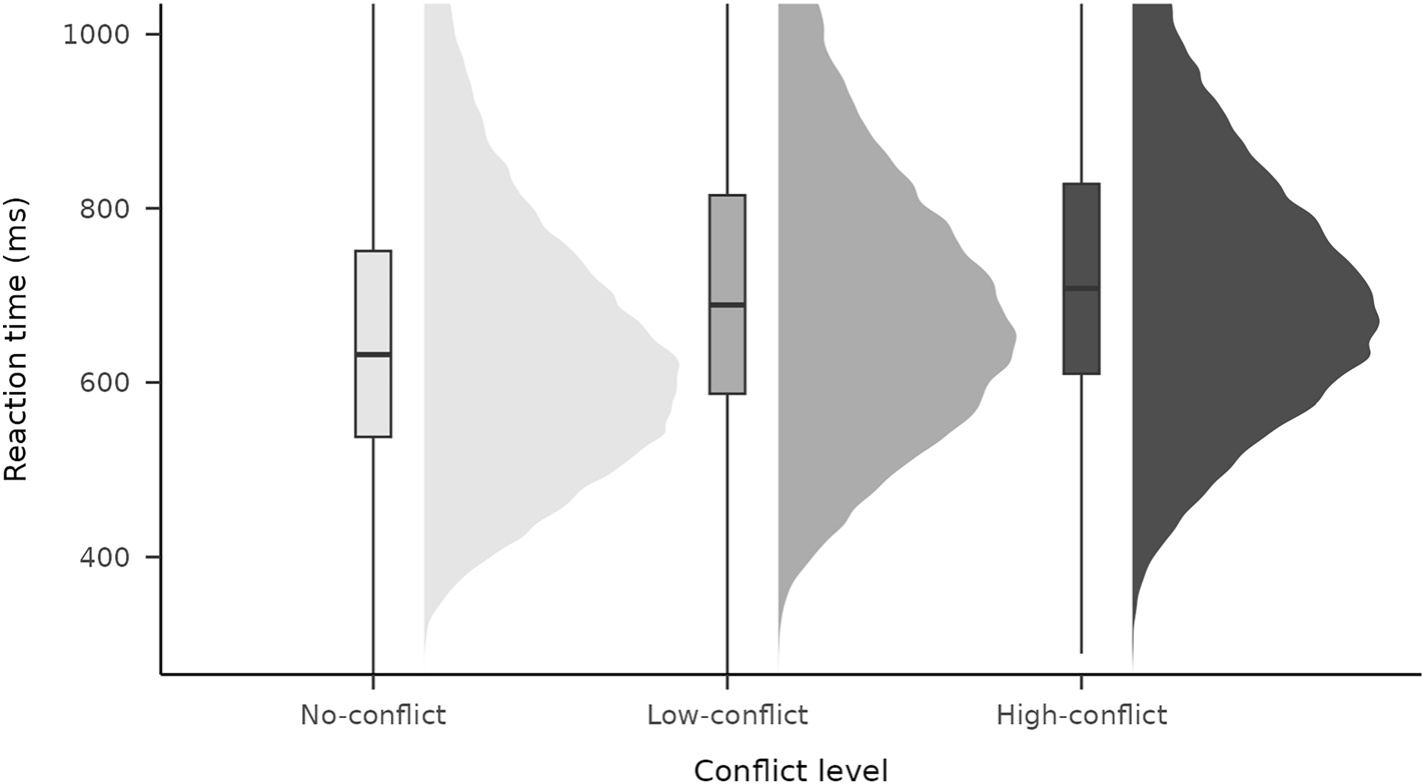
Reaction time for different levels of conflict in the current trial.

First of all we analysed the main effect of congruency without separating low- and high-conflict trials. We have found a significant difference between the RT of congruent and incongruent trials, *F*(1,344) = 2066.4,* p* < 0.001. The mixed-effects linear regression analysis resulted in conclusive evidence for the congruency effect, where the estimated effect was 63.17 ms (SE = 0.77), B_H(0,29.5)_ = Infinite, RR_B > 3_ = [3.46, 18546]. When conflict levels were separated, a significant difference between conflict levels was found in RT (see Fig. 3), *F*(2,688) = 1308.8,* p* < 0.001. In the mixed-effects linear regression we used Helmert contrasts by comparing every level to the level preceding it, NC being the baseline. This analysis also revealed evidence for conflict level effect between NC and LC: estimate = 27.25 ms (SE = 0.47), B_H(0,29.5)_ = Infinite, RR_B > 3_ = [0.98, 9066]; and between LC and HC: estimate = 14.88 ms (SE = 0.3), B_H(0,29.5)_ = Infinite, RR_B > 3_ = [0.38, 5293].

### Sequential congruency effects

**Figure 4:**
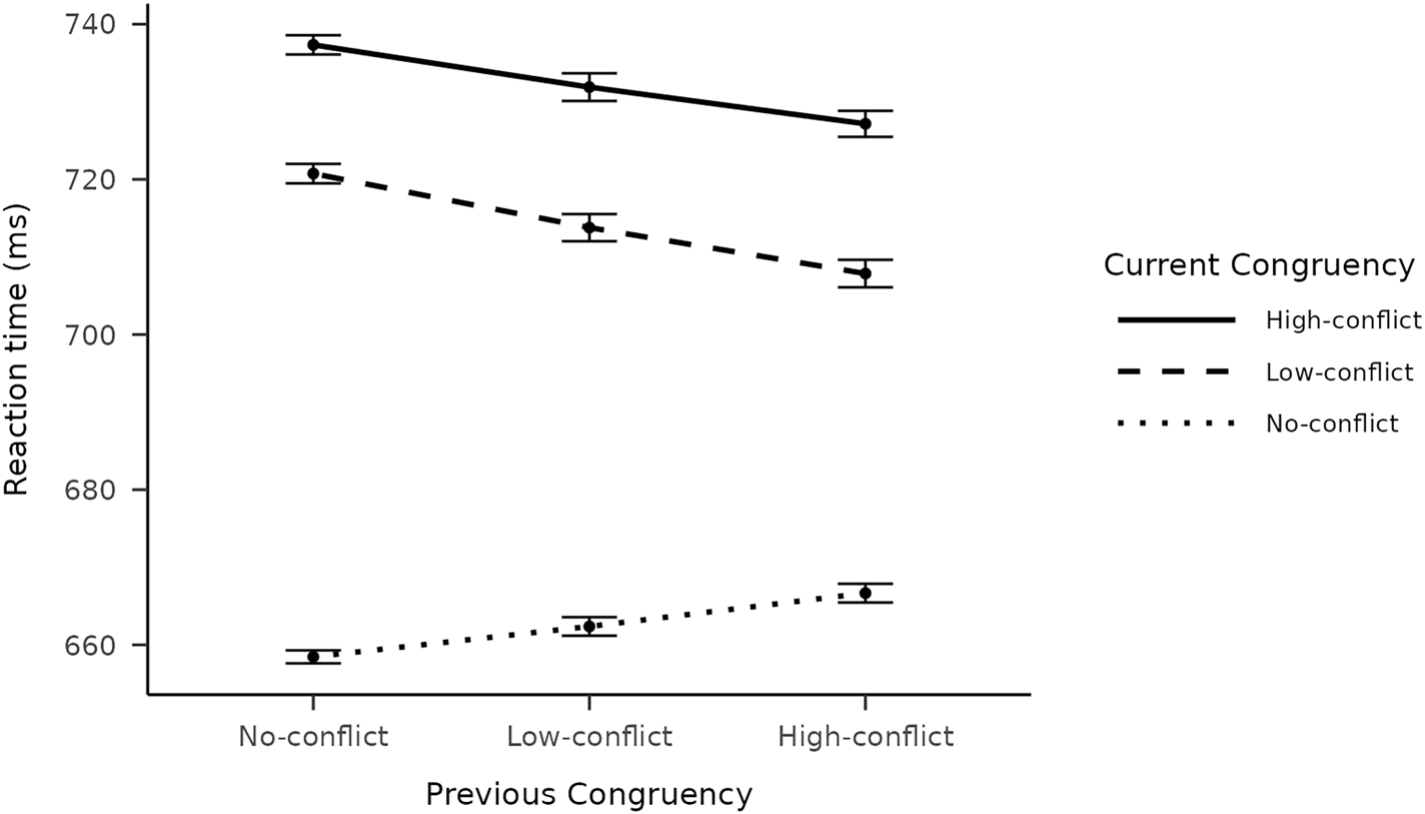
Interaction between previous and current trial congruency type. Mean RT-s on current congruency × previous congruency. Error bars represent the standard error of the mean.

The main congruency sequence effect (CSE) was first analysed without separating conflict levels. We found the classic CSE with both repeated measures ANOVA: F(1344) = 78.29, *p* < 0.001, and mixed-effects linear regression: estimate = 14.73 ms, (SE = 1.54), B_H(0,14.75)_ = 1.05*10^19^, RR_B > 3_ = [0.18, 5670]. More importantly, when conflict levels were separated into NC, LC and HC in previous and current trials (see Fig. 4), an interaction between previous and current conflict level was found, F(41376) = 15.487, *p* < 0.001. A mixed-effect linear regression with conflict level coded as a numeric predictor also resulted in conclusive evidence for an interaction between previous and current conflict level: estimate = 5.1 ms (SE = 0.56), B_H(0,7.38)_ = 7.96*10^17^, RR_B > 3_ = [0.07, 1996]. As we cannot assume the level of conflict to be a continuous parameter, we created another mixed-effect linear regression model in which the level of conflict was coded as a categorical predictor using Helmert contrasts, to investigate the interaction by comparing every level to the level preceding it, NC being the baseline.

Using Helmert contrasts also allowed us to differentiate between the two predictions provided by the repetition expectancy theory and the extended conflict monitoring theory models. The different effect patterns predicted by the two theories posit different hypotheses in regard to specific contrasts in the data (see Fig. 1 for a visualisation), most importantly:

- NC and LC previous trials × LC and HC current trials: the repetition expectancy theory predicts that the LC-HC difference on the current trial will increase following LC trials, while the extended conflict monitoring predicts that this difference, if anything, will decrease.
- LC and HC previous trials × NC and LC current trials: the repetition expectancy theory predicts that the NC-LC difference on the current trial will increase following HC trials, while the extended conflict monitoring predicts that that difference will decrease.

We used normal distributions as priors in these analyses, because we did not assume the direction of the interactions, as different theories predict different interaction patterns. We have found no interaction between the contrast of NC and LC previous trials × the contrast of LC and HC current trials: estimate = 0.49 ms, (SE = 0.37), B_N(0,14.75)_ = 0.06, RR_B <1/3_ = [2.69, 99901], where conclusive evidence for the null hypothesis was found.

We have found conclusive evidence for an interaction between the contrast of LC and HC previous trials × the contrast of NC and LC current trials with the pattern being for an NC–LC difference *reduction* following HC trials: estimate = 2.64 ms, (SE = 0.37), B_N(0,14.75)_ = 2.56*10^9^, RR_B > 3_ = [9.92,975].

Furthermore Bayesian test between the contrast of LC and HC previous trials × the contrast of LC and HC current trials resulted in anecdotal, but inconclusive evidence for an interaction with the pattern being for a slight LC-HC difference increase following HC trials: estimate = 0.73 ms, (SE = 0.23), B_N(0,14.75)_ = 2.02, RR_B > 1/3_ = [9.92,89]. We have found conclusive evidence for an interaction between the contrast of NC and LC previous trials × the contrast of NC and LC current trials with the pattern being for an NC-LC difference decrease following LC trials: estimate = 2.66 ms, (SE = 0.57), B_N(0,14.75)_ = 2012, RR_B > 3_ = [0.2, 837].

## Discussion

In this study we replicated the experimental design of Zhang and colleagues^11^ on a sizable and diverse sample, with the goal to further investigate the effect of parametric manipulation of distractors on adaptation of control. We found that increasing the number of incompatible distractors around a target indeed leads to slower response times, which suggests an increase in the conflict level of such trials. Secondly, we tested whether there is an interaction between previous and current conflict level and found evidence for the previous conflict level affecting the current conflict level. Further investigations also showed that overall conflict level differences were smaller following higher levels of conflict trials. These results suggest that increasing the level of conflict indeed triggers adaptation of control, and furthermore, greater levels of previous conflict lead to stronger decreases in congruency effects in a monotonic way.

These findings are generally in line with the conclusion of both Forster and colleagues^8^ and the currently replicated study^11^ that tested the impact of conflict on the current trial as a function of the conflict level on the previous trial. In the original study some results however suggested that the effect of the relationship between current and previous conflict on RT might not be monotonic: For example, they found that the RT on LC trials when preceded by LC trials was faster than when preceded by HC trials. This pattern was replicated by the original authors in a newer study^12^ which led them to the conclusion that it is not conflict level per se, but the expectancy of the previous conflict type that is responsible for such adaptive behaviour^3^. This conclusion implied that the model of conflict monitoring is not sufficient to explain congruency sequence effects and such effects in general are not the product of dynamically adjusted control, but the mere expectation of the repetition of the previous trial type. The experimental design let us compare the different predictions of two accounts: the extended conflict monitoring account and the repetition expectancy account. The extended conflict monitoring account considers conflict level as a continuous parameter that informs the monitoring system about the amount of control needed to overcome conflict on the subsequent trial, this way creating a linear relationship between conflict strength and adaptive control. The repetition expectancy account however considers conflict strength type as a discrete type of information that can either enhance performance, when repeated as expected, or impair it, when it is not repeated. The two mechanisms however can be present in conflict resolution at the same time, affecting adaptive behaviour in different ways^38^. To investigate the two predictions, we analysed multiple contrasts: the interaction between NC and LC previous trials × LC and HC current trials. Repetition expectancy predicts an interaction with a greater LC-HC difference after LC compared to NC trials (see Fig. 1, top graph). The extended interpretation of conflict monitoring in contrast predicts, if anything, a slightly smaller LC-HC difference after LC trials compared to NC trials (see Fig. 1, bottom graph). Here we have found evidence for the null hypothesis, that indicates that changes in the level of conflict on previous trials affect both HC and LC trials in a similar way, regardless of repetitions in conflict types. We also analysed the interaction between LC and HC previous trials × NC and LC current trials. Repetition expectancy here predicts an increase in the NC–LC difference when preceded by HC trials compared to LC trials (see Fig. 1, top graph). In contrast, conflict monitoring predicts a decrease in the NC-LC difference after HC trials compared to LC trials (see Fig. 1, bottom graph). Here we have found conclusive evidence for a positive interaction, with decreased congruency effect after HC trials compared to LC trials, which is in line with the conflict monitoring theory’s prediction.

The above results are mostly in line with the extended conflict monitoring account, as congruency effects showed to be in a monotonic relationship with previous trial conflict strength. These results resonate with those of Jiménez and Méndez^39,40^, who, in two studies, investigated a potential repetition expectancy effect in 4-choice Stroop tasks, where the expectancy of subsequent trials was also measured. They have found that congruency effects do not rely on explicit expectancies. Our design did not measure expectancies, but the predictions for the repetition expectancy account relied on the assumption that a repetition is always expected. As our results provided no support for that account, they are in line with the conclusions of Jiménez and Méndez^39,40^.

While we have found patterns that are largely consistent with the extended conflict monitoring theory’s prediction, we did not find stronger adaptation effects (i.e., reduced RTs with higher previous conflict level) on HC trials than on LC trials (in fact, if anything, the LC-HC difference tended to be larger following HC trials). A viable interpretation of this pattern is that conflict monitoring of previous trials may be continuous, but it may not lead to greater conflict adaptation on higher current trial conflicts. Most importantly, regardless of explanatory theories, this study provides further evidence in favour of the notion that adaptive control is not driven by an all-or-none mechanism, but rather a continuous function, depending on the scale of the previous conflict.

While the differences in RTs on different incompatibility levels can be interpreted as changes in the level of conflict, we cannot be sure that it is conflict level that is responsible for the modulations in the magnitude of the congruency effect. In a recent study, we found decreased congruency effects on higher-difficulty conflict trials, where difficulty reflected subjective ratings, as opposed to objective measures of performance such as RT^41^. Several theories are being debated in the literature, such as the affective signaling framework^42–44^, that suggests that it is not conflict itself, but the affect triggered by conflict that drives adaptation of control. According to the affective signaling theory, conflicting stimuli induce negative emotional states, and adaptive patterns in subsequent trials are the product of affective regulation with the goal to decrease the level of negative affect created by trial incongruency. Several studies have found that by intermixing conflict tasks with affective stimuli, the valence or arousal of such affective signals can modulate the strength of conflict adaptation^45–47^. The decrease in congruency effects in our data therefore may be attributed not solely to the level of conflict but rather to the heightened negative affect induced by increased distractor incompatibilities. On the other hand, there is a more general interpretation of adaptive control in conflict tasks, namely the expected value of control theory, that models adaptive control as the function of the value of control, which can be modulated by manipulating the cost or payoff of the effort^48^. According to expected value of control, as task difficulty rises, maintaining the same level of control diminishes task success; thus, to optimise expected control value, control allocation should increase until a threshold where the benefits no longer outweigh associated costs at extremely high task difficulty. In Bognar et al.^41^, we have tested this theory by manipulating task difficulty similarly to the current design and have found an inverted U-shape relationship between perceived task difficulty and control adaptation, where adaptive control increased with task difficulty until a certain point, where it was too high, and adaptation started to decrease. The pattern found in the present study is consistent with the lower difficulty findings of Bognar et al.^41^ in that the decrease in the congruency effect followed a steady line with the increase of difficulty (or conflict level). However, in the current study there was no turning point in congruency effect, perhaps because the difficulty was not increased to an extreme level, where the value of control could reach a turning point. If the current study had involved higher levels of conflict, a U-shaped pattern might have been also observed.

An important limitation of both the current study and the replicated study, is implementing low-conflict trials as the middle option between a classic congruent and incongruent flanker stimulus (i.e., using two rather four incongruent flankers). By this choice, we did not increase but moderated the level of distraction by incompatibility, which made the overall scaling of interference effects dependent on the classic congruency effect between high-conflict and no-conflict trials. If this congruency effect had not turned out to be large enough, the differences between the three levels might not have been observable. This overall small margin in the possible effects between conditions potentially reduces the design’s sensitivity to our manipulation. A more ideal choice of distractor incompatibility levels would be a no-conflict, high-conflict, and higher-conflict combination, where the classic flanker effect is observable between no-, and high-conflict conditions, while a comparable difference between high-, and higher-conflict is also present.

To conclude, we replicated the experimental design of Zhang and colleagues^11^ in a multisite study, with a sizable and diverse sample. We did not fully reproduce the result pattern observed in the original study that was interpreted as providing evidence for the repetition expectancy theory. However, we have found that changes in the level of conflict lead to decreases in congruency effects, moreover the level of previous conflict predicts the amount of such congruency effect decreases. This finding is consistent with the extended interpretation of the conflict monitoring account, and with several related accounts such as the affective signaling and expected value of control framework as well.

## Data Availability

The datasets generated and analysed during the current study are available in the Open Science Framework repository, https://osf.io/rsuzg. This study was preregistered on Open Science Framework: https://osf.io/cfjgz. Aside the anonymized data, experiment code, and analysis code can be also found on the OSF repository of this study: https://osf.io/e5ryf/.
